# Genome Sequence of Methicillin-Resistant *Staphylococcus pseudintermedius* Sequence Type 233 (ST233) Strain K7, of Human Origin

**DOI:** 10.1128/genomeA.00310-13

**Published:** 2013-06-20

**Authors:** Jung-Ho Youn, Arshnee Moodley, Yong Ho Park, Chihiro Sugimoto

**Affiliations:** Division of Collaboration and Education, Research Center for Zoonosis Control, Hokkaido University, Kita-ku, Sapporo, Hokkaido, Japana; Department of Veterinary Disease Biology, University of Copenhagen, Frederiksberg C, Copenhagen, Denmarkb; Department of Microbiology, College of Veterinary Medicine, BK21 Program for Veterinary Science, Seoul National University, Seoul, Republic of Koreac

## Abstract

We report the genome sequence of the methicillin-resistant *Staphylococcus pseudintermedius* strain K7, isolated from the nares of a veterinarian in Seoul, South Korea.

## GENOME ANNOUNCEMENT

*Staphylococcus pseudintermedius* is a commensal in dogs and an important opportunistic pathogen commonly associated with skin and ear infections. *S. pseudintermedius* is not part of the normal human microflora, and occurrence in humans is limited mainly to individuals who have regular contact with pets, such as pet owners and veterinarians. Methicillin-resistant *S. pseudintermedius* (MRSP) is a serious veterinary problem due to its characteristic multidrug-resistance phenotype (1), and it can be transmitted to owners and veterinarians (2, 3). A human MRSP infection has been reported as the result of exposure to a colonized dog (4).

MRSP strain K7, belonging to *spa* type t06 and sequence type 233 (ST233), was isolated from the nares of a healthy female veterinarian at a small animal veterinary teaching hospital in Seoul, South Korea, in April 2008 (1). This strain was determined to harbor the antimicrobial resistance genes *ant(6′)-Ia-aph(3′)-III* (aminoglycoside resistance), *mecA* and *blaZ* (β-lactam resistance), *erm*(B) (resistance to macrolides, lincosamides, and group B streptogramins), *lnu*(A) (lincosamide resistance), *tet*(M) (tetracycline resistance), and *dfrG* (trimethoprim resistance), and the exfoliative toxin gene set.

The genome of K7 was determined using 454 pyrosequencing on a GS Junior genome sequencer at 454 Life Sciences (Roche Diagnostics, Basel, Switzerland), generating 101,942 reads with an average length of 437 bp. *De novo* genome assembly using Geneious v6.1.3 produced 77 contigs of >1,000 bp, with a mean length of 37,767 bp. Automated annotation of genes was performed on the Rapid Annotations using Subsystems Technology (RAST) server (5).

The draft genome of K7 is estimated to be 2,763,498 bp and has a G+C content of 37.4%. K7 has a slightly lower G+C content (0.2 to 0.8%) than the other sequenced *S. pseudintermedius* strains, MRSP strain E140 (accession no. ANOI01000001) and the two methicillin-susceptible *S. pseudintermedius* strains HKU 10-03 (accession no. CP002439) and ED99 (accession no. CP002478). The K7 genome is 6 kb smaller than that of MRSP E140 but is 146 and 191 kb larger than those of HKU 10-03 and ED99, respectively. Methicillin resistance is associated with the presence of staphylococcal cassette chromosome *mec* element (SCC*mec*) type V (28 kb). K7 is predicted to have 2,937 protein-coding sequences and 85 tRNAs.

This K7 MRSP genome of human origin will facilitate future comparative genomic analyses of *S. pseudintermedius* strains from different countries, as well as aid in the identification of factors related to host range.

### Nucleotide sequence accession numbers.

The K7 genome has been deposited at DDBJ/EMBL/GenBank under the accession no. BARM01000001 to BARM01000077.
